# Low handgrip strength with asymmetry is associated with elevated all-cause mortality risk in older Chinese adults with abdominal obesity

**DOI:** 10.1371/journal.pone.0306982

**Published:** 2024-08-13

**Authors:** Ling Wei, Binyou Wang, Yilin Wang

**Affiliations:** Department of Psychiatry, Zigong Mental Health Center, the Zigong Affiliated Hospital of Southwest Medical University, Zigong, Sichuan Province, China; Ehime University Graduate School of Medicine, JAPAN

## Abstract

**Background and objectives:**

Low handgrip strength (HGS) and abdominal obesity (AO) have been reported to be linked to an increased all-cause mortality risk in older adults. However, the combined impact of AO with low HGS and/or HGS asymmetry on mortality risk remains unclear. Therefore, this study aimed to investigate the synergistic effects of AO and abnormal HGS on mortality risk among Chinese older adults.

**Methods:**

Baseline data of the China Health and Retirement Longitudinal Study in 2011, along with mortality outcomes obtained in 2018 were used for the analysis. Low HGS was identified as HGS <18 kg in women or <28 kg in men, while HGS asymmetry is defined as an HGS of either hand > 10% stronger than the other. AO was characterized by a waist circumference ≥90 cm in men and ≥85 cm in women. Logistic regression analysis was used to evaluate the relationship between AO, abnormal HGS and mortality risk.

**Results:**

A total of 5186 subjects aged 60 years or older were enrolled, 50.6% of whom were male. The proportions of participants with only AO, only low HGS, only HGS asymmetry, low HGS with asymmetry, both AO and low HGS, both AO and asymmetric HGS, and AO with both low HGS and asymmetry were 20.0%, 6.1%, 16.6%, 8.3%, 3.2%, 13.4%, and 3.9%, respectively. Over the course of a 7-year follow-up interval, 970 of these individuals died, with 13.4%, 12.4%, 13.6%, 15.5%, 4.1%, 10.1% and 6.9% of deaths in the above groups, respectively. The adjusted logistic regression analysis model confirmed that only low HGS (OR = 1.897, 95%CI: 1.386–2.596, *p*<0.001), low HGS with asymmetry (OR = 1.680, 95%CI: 1.265–2.231, *p*<0.001), and AO combined with both low HGS and asymmetry (OR = 2.029, 95%CI: 1.381–2.981, *p*<0.001) were associated with a higher risk of mortality.

**Conclusions:**

Low HGS, with or without asymmetry, is associated with increased mortality risk in older Chinese adults without AO, and the combination of low HGS and HGS asymmetry further elevates mortality risk in those with AO.

## Introduction

Abdominal obesity (AO) is a type of obesity in which fat accumulates mainly in the abdomen [1], and it is commonly seen in older adults [2]. Although numerous studies have linked AO with increased frailty and all-cause mortality [2–5]. However, this relationship may be more complex in older people, and the relationship between all-cause mortality and AO remains controversial. For instance, using data from 2017 community-dwelling adults (≥ 65 years) followed for 17 years, Alharbi et al. [4] found a significant association between AO and increased all-cause mortality. However, this was not confirmed by Kuk et al. [6] and, furthermore, an analysis of 21,109 adults in the USA by Lo et al. [7] found that AO was associated with a reduced risk of all-cause mortality and that AO was particularly protective in older women over 65 years of age. These conflicting results indicate a clear need for further research on the risk of all-cause mortality in older adults with AO.

Aging commonly results in diminished muscle function, characterized by reduced muscle mass and strength [8]. This decline has significant implications for overall health and the capacity to engage in daily activities [9]. Handgrip strength (HGS) has emerged as a robust biomarker for evaluating muscle strength [10], with low HGS serving as an indicator of compromised muscle strength [11]. Low HGS is a widespread phenomenon among older adults globally, with reported prevalence rates of approximately 31.0% in Americans [12], 32.6% in South Koreans [13], and 28.0% in Chinese [14]. Recent studies have highlighted a strong association between low HGS and a range of adverse health outcomes, including mental health issues [15], the development of chronic diseases [16], and increased mortality rates [17]. A significant study by Alexandre et al. [18] conducted in the UK and Brazil, revealed that older adults with AO compounded by low HGS faced the highest risk of all-cause mortality over ten years, underscoring the critical importance of screening for low HGS in this population. However, this research focused on the relationship between HGS and mortality, specifically in the older population with AO in the UK and Brazil, suggesting that the existence of a similar relationship across diverse regions, demographic profiles, and cultures demands further exploration. It is crucial to recognize the discrepancies in criteria for defining low HGS: Alexandre et al. used thresholds of <26 kg for men and <16 kg for women, while the Asian Working Group for Sarcopenia (AWGS) 2019 criteria are <28 kg for men and <18 kg for women [19]. This discrepancy underscores the need to validate the relationship between HGS and mortality within older AO populations in regions such as China, where different demographic contexts, cultural norms and distinct ‘low HGS’ identification criteria exist.

Moreover, beyond the low HGS, HGS asymmetry is a significant indicator of compromised muscle function [8, 20]. This condition is also notably prevalent among the older population and is reported to be fairly prevalent worldwide: about 53.5% of Americans [21], 45.6% in South Koreans [22], and 45.2% in Chinese [14]. Defined as an HGS of either hand being >10% stronger than the other [19], HGS asymmetry has been associated with an increased risk of psychiatric disorders [14], cardiovascular disease [23], chronic morbidity status [24], falls [21] and mortality in older adults [8, 25]. However, research examining the relationship between HGS asymmetry and all-cause mortality in older adults with AO has yet to be fully explored.

HGS asymmetry may precede low HGS [20], and the co-occurrence of low and asymmetric HGS implies more severe muscle dysfunction, exacerbating the risk of adverse health outcomes in the aging process [10, 23, 26, 27]. Therefore, it is hypothesized that a synergistic effect of low HGS and/or HGS asymmetry may influence the risk of death in older Chinese AO patients. In this study, the older Chinese population was categorized into groups based on only low HGS, only HGS asymmetry, the combination of low HGS and HGS asymmetry, and only AO and its combination with low HGS and/or HGS asymmetry. This study aims to investigate whether these HGS abnormalities are associated with all-cause mortality in older Chinese adults with AO and to quantify the extent of this association among the defined groups.

## Methods

### Participant data

This present study was based on data obtained from individuals 45+ years of age from the 2011–2018 waves of the ongoing longitudinal China Health and Retirement Longitudinal Study (CHARLS). The CHARLS questionnaire consists of eight modules: (a) Household Registration Form, (b) Basic Information, (c) Household, (d) Health Status and Functioning, (e) Health Care and Insurance, (f) Work, Retirement, and Pensions, (g/h) Income, Expenditures, and Assets, (i/j) Housing Characteristics, and Interviewer Observation. All interviewers underwent professional training before administering the questionnaires. Ethical approval for this project was granted by the Biomedical Ethics Review Committee of Peking University (IRB00001052-11015) and the need for informed consent for the present study was waived by the Ethics Committee of Zigong Mental Health Center due to the publicly accessible nature of these data (IRB number: 2023041002). The data were initially collected from 17,596 individuals in 2011 (Wave 1). The inclusion criteria in the present study were: (1) being over the age of 60 years; (2) having accurate and valid measurements of waist circumference; (3) having valid and correct HGS data for both hands; (4) having follow-up data over seven years. Participants were excluded if they did not meet these criteria, if any of the corresponding data were incorrect, or if they were lost to follow-up. Finally, a total of 5186 participants over the age of 60 were enrolled.

### Assessment of abnormal handgrip strength and abdominal obesity

Before beginning the HGS assessment, the investigator demonstrated the correct posture and technique, ensuring participants fully understood the procedure. The participants in the study underwent HGS measurements in different positions: standing or sitting with assistance if they were unable to stand independently. In both cases, their elbows were flexed at a 90-degree angle. The evaluations commenced with the non-dominant hand, followed by the dominant hand, and this sequence was repeated twice. Participants were given adequate breaks between each round of measurements to ensure they felt prepared before proceeding to the next round. As per the consensus criteria of the AWGS 2019 [19], the highest value from the dominant hand was used as the final HGS value. Low HGS was classified as a maximum HGS of the dominant hand < 28 kg for males or < 18 kg for females. HGS asymmetry was defined as an HGS of either hand > 10% stronger than the other [25].

The waist circumference of each participant was measured while standing upright, using a measuring tape positioned at the umbilical level during a calm exhalation. AO was defined as a waist circumstance ≥90 cm in men and ≥85 cm in women [28].

In this study, the participants were classified into eight groups. These groups included a normal group and seven abnormal groups. The abnormal groups were classified based on specific attributes such as low HGS only, asymmetric HGS only, AO only, asymmetric and low HGS, AO and low HGS, AO and asymmetric HGS, and a combination of low HGS and HGS asymmetry with AO. These classifications allowed for a better understanding of different conditions within the study population.

### Mortality outcomes

The seven-year mortality outcome data for these patients were obtained from the 2018 sample information dataset.

### Covariates

Participant covariate information was retrieved from the baseline of the first wave (2011) CHARLS survey. This included information on sex, age, body mass index (BMI), sleep duration, marital status (married, separated or divorced or single), education level (illiterate, primary school or lower, junior high school or higher), left chest pain, chest pains when climbing stairs/uphill or walking quickly, physical disability, presence of physical disabilities, brain damage or mental retardation, visual impairments, auditory impairments, speech impediments, hypertension, dyslipidemia, diabetes, history of cancers or other malignancies, chronic lung conditions, liver disease, heart problems, history of stroke, kidney disease, digestive disorders, emotional or psychiatric issues, memory-related diseases, asthma, arthritis or rheumatism, history of falls, smoking habits, and history of alcohol consumption.

In order to determine the frequency of chronic illnesses, participants were queried about the diagnoses of specific ailments provided by medical professionals. The comprehensive list of these health conditions included dyslipidemia, high blood pressure, diabetes or high blood sugar levels, cancer or malignant growths (excluding non-life-threatening skin cancers), liver ailments (excluding fatty liver, growths, and cancer), chronic respiratory diseases like chronic bronchitis and emphysema (excluding conditions associated with growths or cancer), heart-related problems such as heart attack, angina, congestive heart failure, coronary artery disease, and other cardiac issues, stroke, kidney problems (excluding conditions linked to growths or cancer), gastrointestinal disorders (excluding conditions related to growths or cancer), memory-related illnesses like Alzheimer’s disease and Parkinson’s disease, brain degeneration, as well as conditions such as rheumatism and arthritis.

### Statistical analyses

Data were analyzed with SPSS 25.0, with a two-sided *p*< 0.05 being considered significant. Non-normally distributed survival data were reported as medians (P25, P75), while categorical data were reported as numbers with percentages. Baseline characteristics were compared using rank-sum tests and Pearson chi-squared tests.

Possible relationships between AO, abnormal HGS and seven-year follow-up mortality among enrolled subjects were explored with logistic regression analyses. Model 1 was unadjusted. Covariates that were significantly related to mortality (*p* < 0.05) were used to adjust Model 2, and included sex, age, BMI, education, marital status, left-chest pain, chest pains when climbing stairs/uphill or walking quickly, brain damage/mental retardation, vision problems, hearing problems, speech impediments, hypertension, dyslipidemia, diabetes, chronic lung diseases, stroke, memory-related disease, arthritis or rheumatism, asthma, smoking history, and drinking history.

## Results

**Fig 1:**
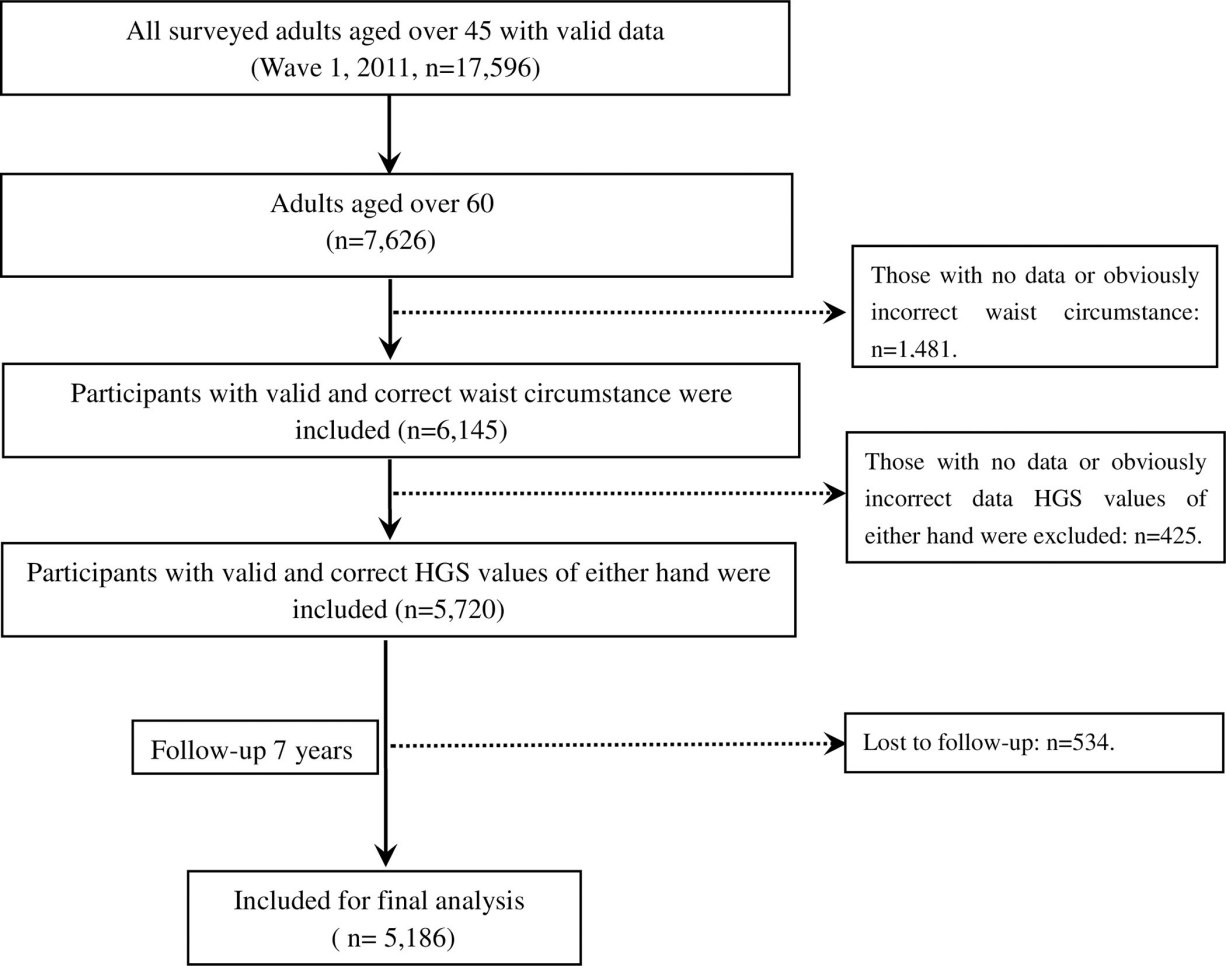
Flowchart of the participant’s selection process. The number of participants excluded or maintained at each step is reported with the related reasons.

The process used to screen participants is shown in Fig 1. Initially, 17,596 adults aged 45 years and above were included in the study. Of these, 7626 were aged 60 years and over; of these 1481 had no data or incorrect data on waist circumference, and 425 had no or incorrect HGS values. Ultimately, a total of 5186 participants over the age of 60 were enrolled, of which 50.6% (2625/5186) were male and 49.4% (2561/5186) were female.

Table 1 presents a summary of the comparative baseline characteristics of the participants together with mortality during seven years of follow-up. During this follow-up period, 970 deaths were recorded, including 589 males (60.7%) and 381 females (39.3%). Relative to the survival cohort, significant differences were observed in these participants in terms of sex (*p*<0.001), age (*p*<0.001), BMI (*p*<0.001), education level (*p*<0.001), marital status (*p*<0.001), left-chest pain (*p* = 0.001), chest pains when climbing stairs/uphill or walking quickly (*p*<0.001), brain damage/mental retardation (*p* = 0.039), vision problems (*p*<0.001), hearing problems (*p*<0.001), speech impediment (*p*<0.001), hypertension (*p* = 0.003), dyslipidemia (*p* = 0.007), diabetes or high blood sugar (*p* = 0.017), chronic lung diseases (*p*<0.001), stroke (*p* = 0.002), memory-related disease (*p* = 0.004), arthritis or rheumatism (*p* = 0.013), asthma (*p*<0.001), smoking history (*p*<0.001), and drinking history (*p* = 0.003). The other covariates showed no significant differences between the two cohorts.

**Table 1:** Baseline characteristics of participants according to the seven years follow-up mortality.

Variable		Survival N = 4216	Died N = 970	*p*-value
Sex, n(%)	Male	2036(48.3)	589(60.7)	<0.001
	Female	2180(51.7)	381(39.3)	
Age,year,median (P25,P75)		65(62,70)	72(66,78)	<0.001
BMI,kg/m2,median (P25,P75)		22.7(20.4,25.3)	21.6(19.4,24.3)	<0.001
Sleeping Time (h),median (P25,P75)		6(5,8)	6(5,8)	0.787
Education, n(%)	Illiterate	1508(35.8)	449(46.4)	<0.001
	Primary school and below	1964(46.6)	412(42.6)	
	Junior high school and above	742(17.6)	106(11)	
Marital status, n(%)	Married	3424(81.2)	654(67.4)	<0.001
	Divorced or Widowed or Single	792(18.8)	316(32.6)	
Left Chest Pain, n(%)	Yes	789(18.9)	219(23.5)	0.001
	No	3391(81.1)	711(76.5)	
Chest Pains When Climbing Stairs/Uphill or Walking Quickly, n(%)	Yes	584(14)	169(18.2)	<0.001
	No	3463(82.9)	710(76.3)	
	Not applicable	130(3.1)	52(5.6)	
Physical Disabilities, n(%)	Yes	151(3.6)	45(4.6)	0.12
	No	4062(96.4)	925(95.4)	
Brain Damage/Mental Retardation, n(%)	Yes	106(2.5)	36(3.7)	0.039
	No	4105(97.5)	933(96.3)	
Vision Problems, n(%)	Yes	342(8.1)	127(13.1)	<0.001
	No	3873(91.9)	842(86.9)	
Hearing Problems, n(%)	Yes	557(13.2)	211(21.8)	<0.001
	No	3657(86.8)	758(78.2)	
Speech Impediment, n(%)	Yes	11(0.3)	14(1.4)	<0.001
	No	4196(99.7)	955(98.6)	
Hypertension, n(%)	Yes	1249(29.7)	335(34.7)	0.003
	No	2950(70.3)	631(65.3)	
Dyslipidemia, n(%)	Yes	420(10.2)	70(7.3)	0.007
	No	3712(89.8)	886(92.7)	
Diabetes or High Blood Sugar, n(%)	Yes	266(6.4)	82(8.5)	0.017
	No	3915(93.6)	883(91.5)	
Cancer or Malignant Tumor, n(%)	Yes	34(0.8)	12(1.2)	0.198
	No	4160(99.2)	954(98.8)	
Chronic Lung Diseases, n(%)	Yes	520(12.4)	208(21.5)	<0.001
	No	3678(87.6)	759(78.5)	
Liver Disease, n(%)	Yes	157(3.8)	41(4.2)	0.473
	No	4028(96.2)	925(95.8)	
Heart Problems, n(%)	Yes	611(14.6)	164(17)	0.06
	No	3579(85.4)	802(83)	
Stroke, n(%)	Yes	104(2.5)	42(4.3)	0.002
	No	4102(97.5)	925(95.7)	
Kidney Disease, n(%)	Yes	275(6.6)	68(7.1)	0.579
	No	3913(93.4)	895(92.9)	
Stomach or Other Digestive Disease, n(%)	Yes	985(23.4)	203(21)	0.101
	No	3219(76.6)	765(79)	
Emotional, Nervous, or Psychiatric Problems, n(%)	Yes	62(1.5)	15(1.6)	0.86
	No	4132(98.5)	950(98.4)	
Memory-Related Disease, n(%)	Yes	77(1.8)	32(3.3)	0.004
	No	4123(98.2)	935(96.7)	
Arthritis or Rheumatism, n(%)	Yes	1628(38.7)	334(34.4)	0.013
	No	2578(61.3)	636(65.6)	
Asthma, n(%)	Yes	197(4.7)	88(9.1)	<0.001
	No	4004(95.3)	879(90.9)	
Fallen Down, n(%)	Yes	819(19.6)	175(18.9)	0.612
	No	3358(80.4)	752(81.1)	
Smoking history, n(%)	Yes	1718(40.8)	525(54.2)	<0.001
	No	2496(59.2)	444(45.8)	
Drinking history, n(%)	No	2665(63.7)	560(58.5)	0.003
	Yes	1516(36.3)	397(41.5)	

Table 2 presents the results of the seven-year follow-up on all-cause mortality, comparing different groups. The proportions of participants with AO only, low HGS only, HGS asymmetry only, low HGS with asymmetry, AO and low HGS, AO and asymmetric HGS, and AO and low HGS with asymmetry were 20.0%, 6.1%, 16.6%, 8.3%, 3.2%, 13.4%, and 3.9%, respectively. Significant differences were observed in survival and seven-year follow-up mortality between all the groups (*p*<0.001).

**Table 2:** Comparison of seven years follow-up all-cause mortality between different groups.

Variable	Survival n = 4216	Died n = 970	*p*-value
Normal,n(%)	1244(29.5)	233(24.0)	<0.001
AO only,n(%)	908(21.5)	130(13.4)	
Low HGS only,n(%)	194(4.6)	120(12.4)	
Asymmetric HGS only,n(%)	729(17.3)	132(13.6)	
Low HGS with asymmetry, n(%)	283(6.7)	150(15.5)	
AO and low HGS,n(%)	125(3)	40(4.1)	
AO and asymmetric HGS,n(%)	596(14.1)	98(10.1)	
AO and low HGS with asymmetry,n(%)	137(3.2)	67(6.9)	

Note: HGS = handgrip strength, AO = abdominal obesity

Table 3 displays the findings from the logistic regression analysis examining the associations between AO, abnormal HGS, and mortality during the seven-year follow-up. Compared with participants with normal function, AO only (odds ratio [OR] = 0.764, 95%CI: 0.607–0.963 *p* = 0.023), low HGS only (OR = 3.303, 95%CI: 2.528–4.314, *p*<0.001), low HGS with asymmetry (OR = 2.830, 95%CI: 2.221–3.606, *p*<0.001), AO with low HGS (OR = 1.708, 95%CI: 1.165–2.505, *p* = 0.006), and AO combined with asymmetric and low HGS (OR = 2.611, 95%CI: 1.889–3.610, *p*<0.001), were significantly associated with mortality risk in older adults (Model 1). In the fully adjusted model (Model 2), significant associations were found between mortality and low HGS only (OR = 1.897, 95%CI: 1.386–2.596, *p*<0.001) and low HGS with asymmetry (OR = 1.680, 95%CI: 1.265–2.231, *p*<0.001). However, AO was only associated with increased mortality in older adults when combined with low HGS and asymmetry (OR = 2.029, 95%CI: 1.381–2.981, *p*<0.001).

**Table 3:** Associations between abdominal obesity, handgrip strength and seven-year follow-up mortality.

Variable	Model 1		Model 2	
	OR (95% CI)	*p*-value	OR (95% CI)	*p*-value
Normal,n(%)	1(Ref)	**-**	1(Ref)	**-**
AO only,n(%)	0.764(0.607,0.963)	0.023	0.886(0.682,1.151)	0.364
Low HGS only,n(%)	3.303(2.528,4.314)	<0.001	1.897(1.386,2.596)	<0.001
Asymmetric HGS only,n(%)	0.967(0.766,1.219)	0.775	0.940(0.730,1.212)	0.635
Low HGS with asymmetry,n(%)	2.830(2.221,3.606)	<0.001	1.680(1.265,2.231)	<0.001
AO and low HGS,n(%)	1.708(1.165,2.505)	0.006	1.272(0.820,1.973)	0.283
AO and asymmetric HGS,n(%)	0.878(0.680,1.133)	0.318	1.063(0.798,1.418)	0.675
AO and low HGS with asymmetry,n(%)	2.611(1.889,3.610)	<0.001	2.029(1.381,2.981)	<0.001

Note: HGS = handgrip strength, AO = abdominal obesity

Model 1: unadjusted model

Model 2: adjusting for sex, age, BMI, education, marital status, left chest pain, chest pains when climbing stairs/uphill or walking quickly, brain damage/mental retardation, vision problems, hearing problems, speech impediment, hypertension, dyslipidemia, diabetes, chronic lung diseases, stroke, memory-related disease, arthritis or rheumatism, asthma, smoking history and drinking history.

## Discussion

This study is the first to examine the relationships between combined AO and low HGS, with or without HGS asymmetry, and mortality risk among older Chinese adults aged 60 and above. The main findings suggested that the coexistence of AO and low HGS with asymmetry in older adults was associated with the increased mortality risk. In addition, a significant interaction was also observed between low HGS alone and low HGS with asymmetry in relation to mortality risk. Therefore, regular measurement of HGS is strongly recommended as an indicator for individuals aged 60 and above in China. Specifically, it is necessary for older adults without AO to screen for low, with or without asymmetric HGS; for older adults with AO, particular attention should be given to low HGS with HGS asymmetry.

To the best of our knowledge, there are no studies that have used the same subgroups, the same duration of follow-up, and similar populations as ours; thus, the following are brief comparisons of the results with those of previous studies. A study by Alexandre et al. [18] investigated whether low HGS combined with AO increased the risk of death among older adults in the UK and Brazil. The study included 6173 older adults over the age of 70 years who were followed up for a mean of 8.3 years and found that 7.2% of the participants had a combination of AO and low HGS, 7.5% had low HGS only, 45% were classified as having AO, and 40.4% were classified as non-HGS/non-AO. The corresponding percentages in the present study were 7.1% (AO with low HGS, AO and low HGS with asymmetric), 14.4% (low HGS only, low HGS with asymmetric HGS), 33.4% (AO only, AO and asymmetric HGS), and 45.1% (normal, asymmetric HGS only). This clearly demonstrates that in the Chinese population there are fewer people with AO and more people with low HGS. The explanation is that the populations and numbers investigated in the two studies differed. Regional differences affect factors such as diet and lifestyle which, in turn, may influence the body weight, fat distribution, and even the prevalence of chronic diseases in the population. Furthermore, the differing criteria for defining low HGS in the two regions may contribute significantly to the observed variations in the prevalence of low HGS.

As people age, the levels of fat, especially abdominal and intermuscular fat, tend to increase, together with changes in the fat distribution [29]. AO has been associated with an increased risk of many health issues, such as heart disease [3, 30], high blood pressure [31], frailty [32], inflammation [33], and certain cancers [34], all of which are associated with all-cause mortality in older adults. Abnormal HGS is associated with an increased risk of neurological and psychiatric disorders [14], cardiovascular disease [35], respiratory disease [36], and cognitive dysfunction [29]. This indicates that both abnormal HGS and AO are associated with all-cause mortality. Furthermore, obesity may also adversely affect muscle function by activating pro-inflammatory cytokines and metabolic abnormalities [37]. Muscle weakness in older adults is associated with reduced metabolism occurring with age, which increases and perpetuates the development of obesity [37]. Thus, AO and abnormal HGS are likely to interact, influencing the risk of all-cause mortality.

The findings of Alexandre et al.’s study suggest that the combination of AO and low HGS poses the highest risk of mortality in older adults in the UK and Brazil [18]. However, this investigation revealed that the combination of AO and low HGS was not a significant risk factor for mortality. The present study found that AO, together with both low and asymmetric HGS, could significantly predict all-cause mortality in older adults in China. There are several possible explanations for these differences. First, the present study classified low HGS as both low HGS only and low HGS with asymmetry. Among the participants with AO, the proportions of those with AO and low HGS with asymmetry and AO with low HGS only were 9.6% and 7.9%, respectively. The proportion of participants with AO and low HGS with asymmetry was observed to be greater in older Chinese adults. Second, the population included in the present study was younger (≥60 years) and the follow-up period may not have been sufficiently long, resulting in a failure to obtain all the death outcomes. Third, different methods and standards used for measuring HGS and AO could account for differences in the HGS and AO values. Based on these results, an increased focus on individuals with low and asymmetric HGS, particularly in those with AO, is strongly recommended due to the significant associations with higher all-cause mortality.

There are multiple limitations to the present study. First, the sample size of participants in each abnormal group was relatively small, potentially leading to a lack of significance in the results. Second, many baseline covariates, including cardiac and liver conditions, relied on self-reports rather than clinical diagnoses, introducing potential subjective biases. Third, the associations between specific causes of death and AO combined with both low HGS and asymmetry were not examined due to insufficient data on the causes of death. Finally, the follow-up period may not have been sufficient to collect adequate mortality outcomes. To further verify the influence of AO and HGS on mortality risk, future research should encompass a broader range of regions and populations, involve extended follow-up periods, and ensure the collection of more rigorously defined baseline data.

## Conclusions

In summary, a significant association was observed between low HGS only and low HGS with asymmetry on the risk of mortality in older Chinese adults, consistent with the findings of previous studies [8, 38]. Meanwhile, low HGS with asymmetry is associated with elevated all-cause mortality risk in older Chinese with AO. Therefore, preventive interventions should prioritize older persons with the above conditions, which may assist in accurately predicting mortality risk and maximize potential public health gains, especially in resource-limited settings.
